# Genome Characteristics of Two Ranavirus Isolates from Mandarin Fish and Largemouth Bass

**DOI:** 10.3390/pathogens12050730

**Published:** 2023-05-17

**Authors:** Xue-Dong Yu, Fei Ke, Qi-Ya Zhang, Jian-Fang Gui

**Affiliations:** 1 College of Fisheries, Huazhong Agricultural University, Wuhan 430070, China; yuxd309027096@webmail.hzau.edu.cn; 2 The Innovative Academy of Seed Design, Institute of Hydrobiology, Chinese Academy of Sciences, Wuhan 430072, China; zhangqy@ihb.ac.cn

**Keywords:** *Siniperca chuatsi* ranavirus, *Micropterus salmoides* ranavirus, complete genome sequence, genome comparison, genome arrangement, functional gene

## Abstract

Ranaviruses are promiscuous pathogens that threaten lower vertebrates globally. In the present study, two ranaviruses (SCRaV and MSRaV) were isolated from two fishes of the order Perciformes: mandarin fish (*Siniperca chuatsi*) and largemouth bass (*Micropterus salmoides*). The two ranaviruses both induced cytopathic effects in cultured cells from fish and amphibians and have the typical morphologic characteristics of ranaviruses. Complete genomes of the two ranaviruses were then sequenced and analyzed. Genomes of SCRaV and MSRaV have a length of 99, 405, and 99, 171 bp, respectively, and both contain 105 predicted open reading frames (ORFs). Eleven of the predicted proteins have differences between SCRaV and MSRaV, in which only one (79L) possessed a relatively large difference. A comparison of the sequenced six ranaviruses from the two fish species worldwide revealed that sequence identities of the six proteins (11R, 19R, 34L, 68L, 77L, and 103R) were related to the place where the virus was isolated. However, there were obvious differences in protein sequence identities between the two viruses and iridoviruses from other hosts, with more than half lower than 55%. Especially, 12 proteins of the two isolates had no homologs in viruses from other hosts. Phylogenetic analysis revealed that ranaviruses from the two fishes clustered in one clade. Further genome alignment showed five groups of genome arrangements of ranaviruses based on the locally collinear blocks, in which the ranaviruses, including SCRaV and MSRaV, constitute the fifth group. These results provide new information on the ranaviruses infecting fishes of Perciformes and also are useful for further research of functional genomics of the type of ranaviruses.

## 1. Introduction

Ranaviruses are members of the genus *Ranavirus* (family *Iridoviridae*) [1], which are nucleocytoplasmic large DNA viruses (NCLDVs). Ranaviruses have been isolated from several poikilotherms, including fishes [2,3,4], amphibians [5,6,7], and reptiles [8]. Several of the poikilotherms are important farmed animals. Thus, ranaviruses represent a great threat to these animals and the related culture industry. The complete genomes of more than 100 ranavirus isolates have been sequenced, including two isolated in our lab, the Rana grylio virus (RGV) and Andrias davidianus ranavirus (ADRV) [6,8,9,10,11,12,13,14,15], which promoted the understanding of virus infection and virus–host interactions. According to the report of the International Committee of Taxonomy of Viruses (ICTV), four genomic phenotypes, frog virus 3 (FV3)-like, Ambystoma tigrinum virus (ATV)-like, common midwife toad virus (CMTV)-like, and Singapore grouper iridovirus (SGIV)-like, has been reported in ranaviruses based on whole genome dot plot comparisons [1], in which RGV was grouped in FV3-like and ADRV was grouped in CMTV-like.

It has been reported that aquaculture has become the fastest-growing agricultural production industry in the world, and a major contributor is China [16,17,18]. Mandarin fish (*Siniperca chuatsi*, also known as Chinese perch) and largemouth bass (*Micropterus salmoides*) are two fishes belonging to the Order Perciformes, which have a delicious taste and high nutrition as food. Thus, the culture of the two fishes has been rapidly developing in recent years in China. It has been reported that the annual production of mandarin fish and largemouth bass in China has been more than between 330 and 600 kilotons in recent years [19]. However, economic losses caused by diseases in these fishes are becoming a serious challenge. One of the important viral pathogens in the aquaculture of the two fishes is the ranavirus, which has been isolated from the two fishes in recent years [20,21,22]. Although there are genome sequences of ranaviruses isolated from the two fishes in the GenBank database, a detailed analysis of the genome architecture and comparison with other ranaviruses are not reported.

In the present study, we isolated a ranavirus from diseased mandarin fish and a ranavirus from diseased largemouth bass. The complete genome of the two ranaviruses was determined. Further genome comparison and analysis revealed the characteristics of the two viruses.

## 2. Materials and Methods

### 2.1. Sample Collection

Diseased largemouth bass and mandarin fish were collected from aquafarms in Hubei province of China from June 2021 to July 2022. Tissues of liver, spleen, and kidney of the diseased fishes were collected for virus isolation.

### 2.2. Virus Isolation

Collected tissues were homogenized in phosphate-buffered saline (PBS) and clarified by centrifugation at 10,000× *g* for 5 min. The supernatants were filtered through a 0.45 μm sterile filter (Millipore, Burlington, MA, USA) and used to infect cell lines.

Different aquatic animal cell lines, Chinese giant salamander thymus cell (GSTC), *Epithelioma Papulosum* Cyprini (EPC), and *Siniperca chuatsi* skin cell (SCSC), which were preserved in our lab, were used in virus isolation and infection. The cells were cultured in M199 medium supplemented with 10% fetal bovine serum (FBS) at 25 °C, except the SCSC cells were cultured in L15 medium with 10% FBS. For virus isolation, monolayers of these cells were inoculated with the above tissue homogenates and incubated at 25 °C. The cells were harvested when advanced cytopathic effects (CPE) were observed, and the supernatant was used for the next round of infection until a stable CPE was obtained. Finally, the infected cells were collected and used as virus stocks after being frozen and thawed. The virus titers were measured by using a 50% tissue culture infectious dose (TCID_50_) assay as described previously [5].

### 2.3. Electron Microscopy

Cells were collected at 48 h post-infection (hpi) by centrifugation at 1000× *g* for 5 min. Cell pellets were pre-fixed with 2.5% glutaraldehyde, followed by post-fixed with 1% osmium tetroxide (OsO_4_), then dehydrated stepwise and embedded in Epon-812. Ultrathin sections were stained with uranyl acetate and lead citrate, and examined with a Hitachi HT-7700 transmission electron microscope (TEM) at 80 KV.

### 2.4. Genomic DNA Extraction and Sequencing

Virus particles were purified from collected infected cells by ultracentrifugation, as described previously [6]. Genomic DNA was extracted from the purified virus particles by using the phenol-chloroform method. Briefly, virus suspensions were mixed with Proteinase K and RNase A (Takara, Dalian, China) and digested in a 56 °C water bath for 30 min. Then, the phenol chloroform isoamyl alcohol solution (25:24:1) was added. After shaking and centrifugation, the top water phase was transferred to a clean EP tube. The DNA was precipitated by 3 M sodium acetate and ethanol and stored at −80 °C for further use.

For genomic DNA sequencing, the insertion libraries were constructed with SMRTbell Express Template Prep Kit 2.0 (Pacific Biosciences, Menlo Park, CA, USA) according to the manufacturer’s instructions and sequenced using a PacBio Sequel II instrument (CCS; The Beijing Genomics Institute, Beijing, China).

### 2.5. Genome Annotation and Analysis

The DNA composition, structure, nucleotide, and amino acid sequences were analyzed with the DNASTAR program (Lasergene, Madison, WI, USA) as described previously [23]. The open reading frames (ORFs) were predicted using SnapGene software (version 6.1.1) and NCBI ORF finder (https://www.ncbi.nlm.nih.gov/orffinder/, accessed on 12 December 2022). The following criteria were considered during ORF prediction: (1) the length was at least 120 bp, (2) the predicted ORF was not located in another larger ORF, (3) overlapping ORFs should have homologs in other sequenced iridoviruses [6]. Comparisons of homologous sequences among different viruses were performed by using BLAST programs (blastn for DNA sequence and blastp for protein sequence). All coding protein sequences of ranavirus were collected from GenBank. Multiple sequence alignments were conducted with ClustalX 1.83, and sequence identities were calculated with the MegAlign program. For a detailed comparison of the ORFs between SCRaV, MSRaV, and other ranaviruses, nine strains of ranaviruses were selected, including the four isolated from mandarin fish and largemouth bass previously and five others representing different genomic types of ranaviruses.

For phylogenetic analysis, the 26 iridovirus core proteins from SCRaV, MSRaV, and other completely sequenced iridoviruses were collected, identified based on homology comparison, and concatenated separately, and a reminder is needed that the Shrimp hemocyte iridescent virus and *Cherax quadricarinatus* iridovirus just have 24 core proteins. The MUSCLE program in Mega software (version 11.0.11) was used to make alignment, and a phylogenetic tree was constructed by the Neighbor-Joining method with default parameters. The Multiple genome alignment, including all 6 isolates from mandarin fish and largemouth bass (SCRaV, MSRaV, mandarin fish ranavirus strain NH-1609, largemouth bass virus strain Alleghany, largemouth bass virus strain GDOU, and largemouth bass virus strain Pine), RGV, FV3, ADRV, CMTV, ATV, epizootic hematopoietic necrosis virus (EHNV), SGIV, and grouper iridovirus (GIV), was performed with the progressive Mauve plugin in Geneious software (version 2023.0.2) [24].

## 3. Results

### 3.1. Virus Isolation and Identification

**Figure 1:**
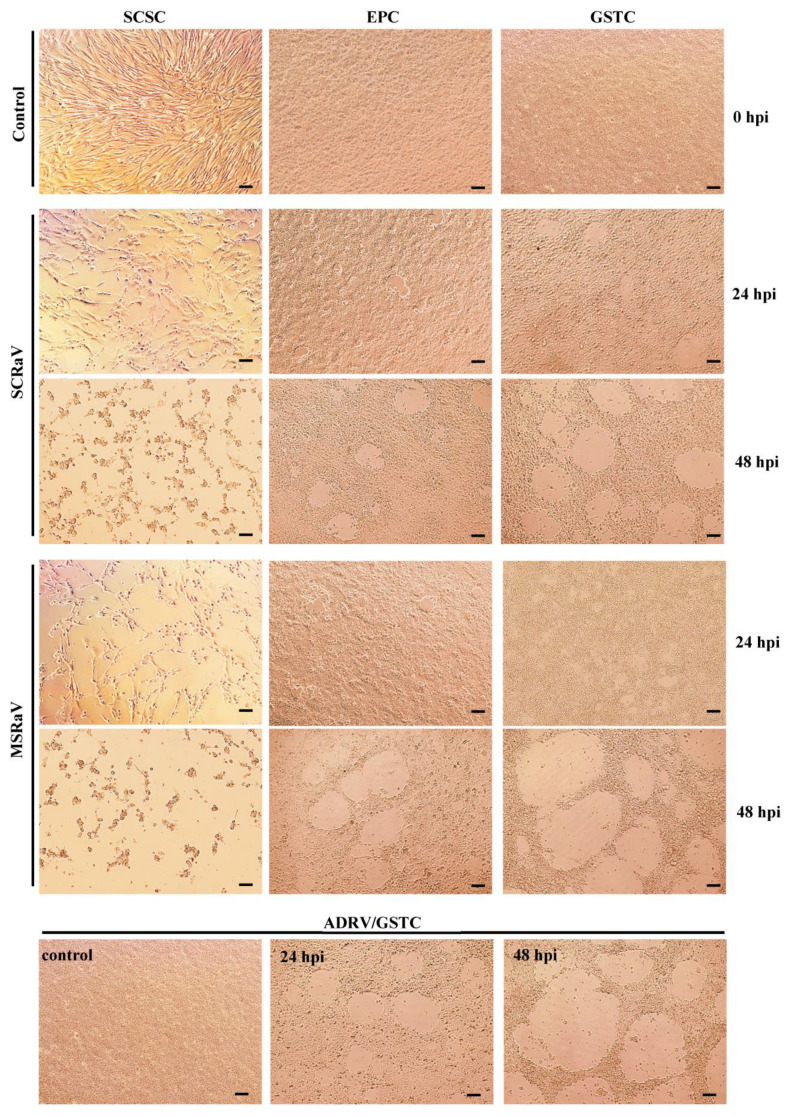
Cytopathic effect caused by SCRaV and MSRaV in SCSC, EPC, and GSTC cells, and ADRV in GSTC cells (ADRV/GSTC) in different time point. Bar = 100 μm.

Tissue extracts from the diseased largemouth bass and mandarin fish both induced cytopathic effect (CPE) in several cultured cells, including SCSC, EPC, and GSTC. Infection of the cells with supernatants from the infected cells still caused typical CPE. A representative CPE in the three cells is shown in Figure 1. The two viruses’ infections both induced the lysis or detachment of cells. In the fibroblast-like SCSC cells, the infected cells lysed or detached rapidly, and only about half of the cells retained at the culture surface at 24 hpi, which formed a discrete distribution. At 48 hpi, most of the SCSC cells have lysed, and the remaining cells became round, indicating their death. For the epithelioid EPC and GSTC cells, a few plaques formed at 24 hpi, and plaques enlarged with infection time due to the lysis and detachment of infected cells. The CPE in SCSC cells seemed more serious than in the other two cells. Infection of GSTC with ADRV, a previously identified ranavirus, was used as a control, which showed similar CPE with the two viruses.

**Figure 2:**
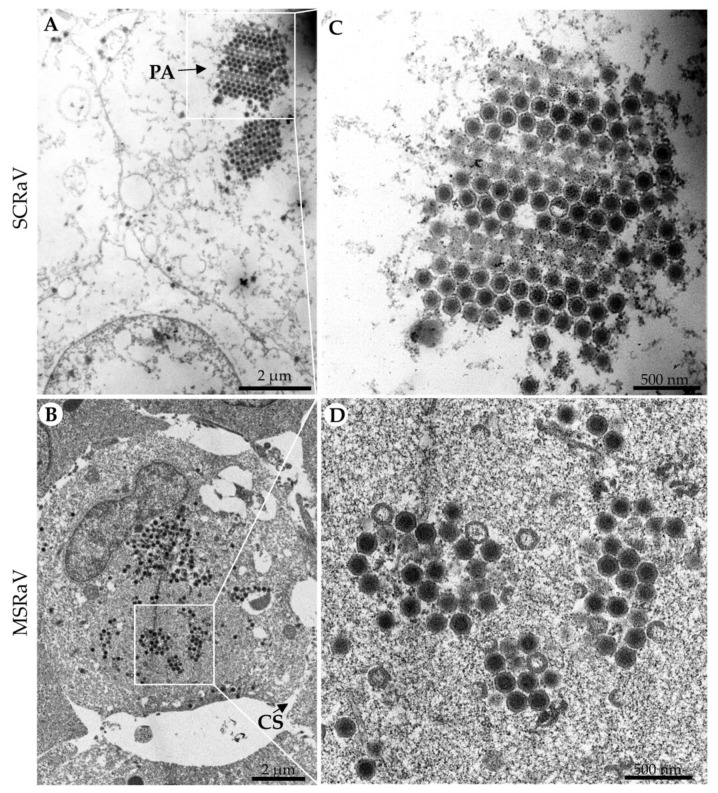
Ultrastructure observation of (**A**,**C**) SCRaV-infected SCSC (48 hpi) and (**B**,**D**) MSRaV-infected GSTC cells (48 hpi). N, nucleus. PA, paracrystalline array. CS, Cell shrinkage.

Ultrastructural observations were performed with SCRaV-infected SCSC cells and MSRaV-infected GSTC cells, respectively. As shown in Figure 2, serious cytoplasmic vacuolation was observed in SCRaV-infected SCSC cells, which caused difficulties in finding cellular organelles (Figure 2A). Cell shrinkage was observed in MSRaV-infected GSTC cells with a compacted and deformed nucleus (Figure 2B). Several regions that were full of mature or immature viral particles can be found in the cells (cytoplasm of GSTC). Intact virions in the ultrathin section are hexagonal or approximately circular, with a diameter of about 160 nm. Paracrystalline arrays that were formed by virion accumulation can be observed in a small number of cells (Figure 2C).

### 3.2. Architecture and General Features of the Two Virus Genomes

The complete genome sequence of the two viruses was determined. The genome of SCRaV consists of 99,405 bp with 105 potential ORFs, and the genome of MSRaV consists of 99, 171 bp with 105 potential ORFs. Detailed information about the predicted ORFs and comparisons with their homologs of other ranaviruses, including the four other ranaviruses (MFRV, LMBV-G, LMBV-A, LMBV-P) isolates from mandarin fish and largemouth bass worldwide were shown in Table 1 and Table S2. The length of the predicted proteins of the two viruses (SCRaV and MSRaV) both ranged from 49 to 1354 aa. There are very high sequence identities between the proteins of the two viruses. Most of their proteins (94/105) have sequence identities of 100% with the homolog. Ten proteins have sequence identities ranging from 92.5% to 99.9% with their homolog. Sequence identity lower than 90% was only obtained in one protein (79L) between the two viruses, which encodes a predicted neurofilament triplet H1-like protein. 

Genome and encoding proteins of SCRaV and MSRaV were then compared with the previously sequenced four ranaviruses from the mandarin fish and largemouth bass worldwide. The results showed that the genome sequence identity between SCRaV and MSRaV was 99.92%, and a range of 98.68–99.88% was obtained between SCRaV and the other four isolates (Table 1). Most of the coding proteins of the six ranaviruses isolated from the two fishes possessed high identities, more than 96% among their homologs. It could be observed that the four isolates from China had higher similarity in genome sequences and coding proteins than the two from the USA (Table S2), especially the six proteins (11R, 19R, 34L, 68L, 77L, and 103R), in which 11R and 68L contain domains of LPXTG-anchored collagen-like adhesin and 77L contains a domain of DNA polymerase III subunit.

However, the sequence identity between the two viruses and ranaviruses from other hosts is not high. Although the sequence identity of the major capsid protein (MCP) between the two viruses and other ranaviruses could reach more than 83%, more than half of the proteins of the two viruses share sequence identity of less than 55% with homologs of ranaviruses from other hosts. There are still several proteins possessing sequence identity lower than 30% (the lowest was 22.3%) with its homolog, and 12 proteins cannot find homologs in iridoviruses from other hosts.

**Figure 3:**
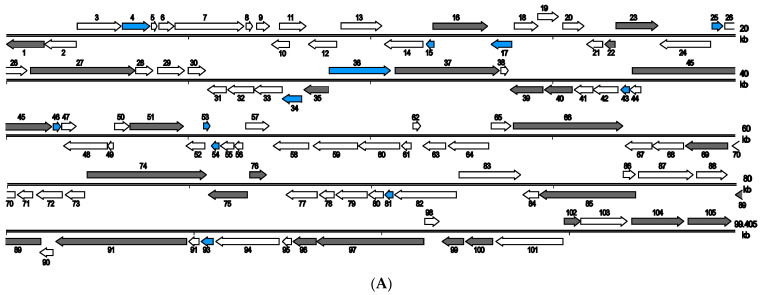

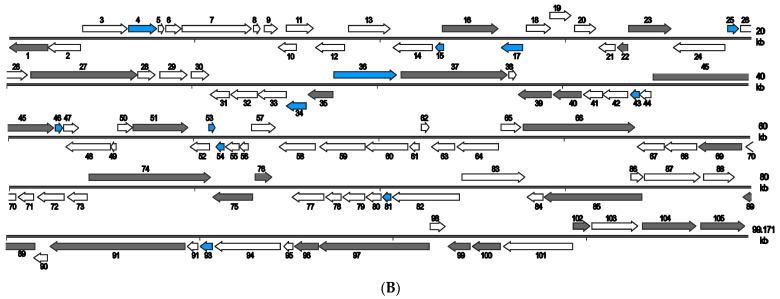
Schematic diagram of the genome organization of SCRaV (**A**) and MSRaV (**B**). The SCRaV and MSRaV genome are 99,405 bp and 99,171 bp in size, respectively, and both contain 105 predicted ORFs. The scale is in kilobase pairs. Arrows indicate the size, location, and orientation of the ORFs. The iridovirus core genes and SCRaV/MSRaV specific genes were shown in black and blue color, respectively. There are 12 SCRaV/MSRaV-specific genes that have no homologs in viruses infecting other animals, including a TNFR-like protein encoded by 17L.

The schematic diagrams of the genome organization of SCRaV and MSRaV are shown in Figure 3. The two viruses have the same genome organization and gene composition. Combined with function analysis, the predicted genes were clustered as genes encoding structural proteins, nucleotide metabolism-related genes, DNA replication- and transcription-related genes, virus–host interaction-related genes, and unknown genes. Detailed information about the genes are described below. Because of the high sequence identity between the two viruses, gene and protein descriptions were mainly performed based on SCRaV.

### 3.3. Structural Proteins

SCRaV *104R* was predicted to encode the major capsid protein (MCP), which contains 463 aa. Among the viral proteins, the MCP of SCRaV and MSRaV has the highest sequence identity with their homologs of ranaviruses infecting other animals. For example, they had a sequence identity of 84% with ADRV MCP and 83.6% with RGV MCP. SCRaV *1L* and *16R* encode two myristylated membrane proteins corresponding to ADRV 2L/RGV 2L and ADRV 58L/RGV 53R, respectively, which belong to core genes of iridoviruses and have been identified as envelope proteins of ranaviruses [25,26]. SCRaV 1L and 16R have sequence identities ranging from 70.5% to 75.2% and 55.4% to 63.7% with their homologs of the last five ranaviruses in Table 1, respectively. There are several other predicted proteins containing transmembrane domain (SCRaV 5R/8R/9R/56L/86R/98R), which could contain envelope proteins.

### 3.4. Nucleotide Metabolism Related Genes

There are 4 predicted proteins that could involve in nucleotide metabolism. SCRaV *71L* encodes a protein of 141 aa, which contains domains of the deoxyuridine 5’-triphosphate nucleotidohydrolase (dUTPase) family. SCRaV 69L (387 aa) and 94L (562 aa) are two homologs of ribonucleotide reductase (RNR) subunit that could catalyze the synthesis of deoxyribonucleotides that was used as precursors of DNA synthesis. SCRaV 99L (189 aa) contains the domain of deoxyribonucleoside kinase (dNK) or thymidine kinase (TK), which is a key enzyme in the salvage of deoxyribonucleosides. The four proteins all have homologs in other ranaviruses.

### 3.5. DNA Replication- and Transcription-Related Genes

For the proteins that could be involved in DNA strand replication, SCRaV *66R* encodes a homolog of DNA polymerase, which has a length of 1004 aa and contains a 3′–5′ exonuclease domain and a B-family DNA polymerase domain. SCRaV *37R* encodes a protein of 955 aa, which contains a domain of primase and the D5_N family. SCRaV 12L (261 aa) is a homolog of the p31K protein of ranaviruses, which has been identified as the virus single-stranded DNA binding (SSB) protein [27]. SCRaV *100L* encodes a protein of 242 aa, whose homologs in other ranaviruses have been considered a homolog of proliferating cell nuclear antigens (PCNA) [28]. In addition, SCRaV 77L (284 aa) contains a domain of DNA polymerase III subunits gamma/tau. SCRaV 82L (566 aa) contains a DNA polymerase III subunit gamma/tau and an SAP domain. SCRaV 75L (356 aa) encodes a putative RAD2 family DNA repair protein, which could be involved in ranavirus DNA recombination and repair [29]. SCRaV 31L (173 aa) contains a domain of Holliday junction resolvases.

For the proteins that could be involved in genome transcription, there are 3 putative subunits of DNA-directed RNA polymerase (RNAP) II. SCRaV *45R* encodes a protein of 1354 aa, which is the putative largest subunit of RNAP (Rpb1). SCRaV 74R has a length of 1094 aa and could be the β subunit of RNAP (Rpb2). SCRaV 28R encodes a protein of 159 aa and contains an RNAP Rpb5 domain. Besides the RNAP subunits, there are possible transcription factors. SCRaV 22L (91 aa) is a transcription elongation factor SII-like protein. SCRaV 40L (253 aa) contains a domain of the poxvirus late transcription factor VLTF3 superfamily. SCRaV 7R (634 aa) contains a domain of transcription termination factor.

In addition, other viral proteins may be involved in genome replication and transcription. For example, SCRaV 48L (400 aa) contains a domain of superfamily II DNA or RNA helicase. SCRaV 97L (949 aa) contains a domain of DEAD-like helicases superfamily and a C-terminal helicase domain of the SNF family helicases.

### 3.6. Virus–Host Interaction Related Genes

Several SCRaV or MSRaV proteins possess domain/motif that has been identified in host proteins, which indicates that these viral proteins could have functions in virus–host interactions. SCRaV *23R* encodes a protein of 385 aa, which contains a domain of the ribonuclease III family. SCRaV 26R (270 aa) is a putative eukaryotic translation initiation factor 2α (eIF-2α)-like protein. SCRaV *41L* encodes a protein of 171 aa containing a domain of the apoptosis regulator proteins of the Bcl-2 family. SCRaV 61L (85 aa) contains a domain of lipopolysaccharide-induced tumor necrosis factor-alpha factor (LITAF). SCRaV 70L (91 aa) contains caspase activation and recruitment domain. SCRaV 72L (237 aa) is a putative tumor necrosis factor receptor (TNFR). SCRaV 95L (79 aa) contains a domain of insulin-like growth factor.

### 3.7. SCRaV- and MSRaV-Specific Genes

Sequence analysis also revealed 12 putative genes (*4R*, *15L*, *17L*, *25R*, *34L*, *36R*, *43L*, *46R*, *53R*, *54L*, *81L*, and *93L*) that no homologs were found for their encoding proteins in viruses of other hosts, which could be considered as specific genes for SCRaV and MSRaV (or SCRaV/MSRaV-like viruses) (Figure 3 and Table 1). It should be noticed that there are 16 genes of SCRaV/MSRaV, including the 12 genes that cannot be found homologs in the compared viruses (ADRV, RGV, FV3, EHNV, and SGIV) in Table 1, but 4 of them (19R, 20R, 44L, and 49L) had homologs in other ranaviruses that were not listed in the table. Most of the specific genes encode hypothetical proteins that no conserved domains/motifs can be found. Only two proteins contain known domains. The 4R protein contains an N-terminal immunoglobulin (Ig)-like domain, and the 17L protein contains a domain of tumor necrosis factor receptor (TNFR), which could be involved in virus–host interactions.

In addition, ORF prediction and analysis also showed that SCRaV/MSRaV encodes five putative proteins (11R, 32L, 33L, 67L, and 68L) that contain domains of LPXTG-anchored collagen-like adhesins. The amino acid length of the 5 predicated proteins is 245, 240, 257, 243, and 288 aa, respectively. Sequence alignment and motif search showed that they all contain variable-length regions full of Gly-X-X repeats, which is a character of LPXTG-anchored collagen-like adhesin. Although homologs of the five proteins could be found in some ranaviruses, the sequence identity between the five proteins and their homologs is low, which made most of their homologs do not contain the LPXTG-anchored collagen-like adhesins domain. So, the five proteins can also be considered SCRaV/MSRaV-specific proteins.

### 3.8. Phylogenetic Analysis

**Figure 4:**
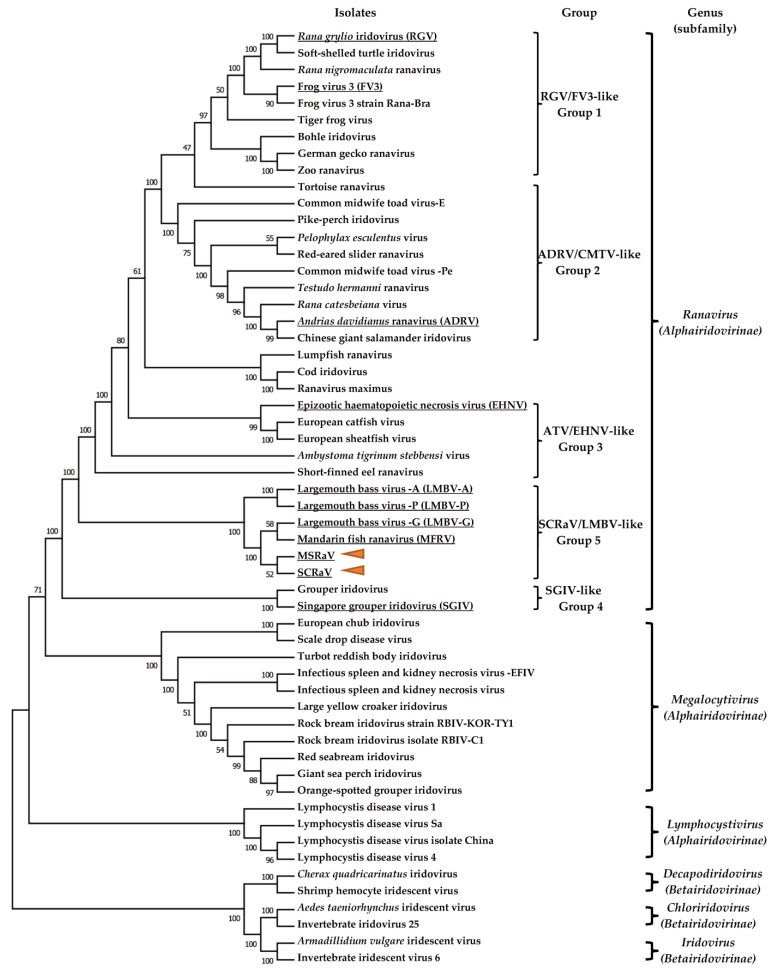
Phylogenetic analysis of the evolutionary relationship among the two ranaviruses and other iridovirus strains based on 26 iridoviral core protein sequences. The two viruses in the present study are indicated by yellow triangles. The ranavirus isolates from mandarin fish and largemouth bass clustered in a clade. The sequences used in the analysis are collected in Table S1.

A phylogenetic tree was constructed based on the proteins of core genes from 56 iridoviruses, including 35 ranavirus isolates (Figure 4). All the ranavirus isolates clustered in a big branch, which could be divided into small branches, including FV3/RGV-like, CMTV/ADRV-like, EHNV/ATV-like, largemouth bass virus (LMBV)/SCRaV-like, and SGIV-like viruses. The two viruses, MSRaV and SCRaV, were clustered with the other largemouth bass virus and mandarin fish ranavirus isolates, which indicated that they belonged to LMBV-like viruses.

### 3.9. Genome Comparison

**Figure 5:**
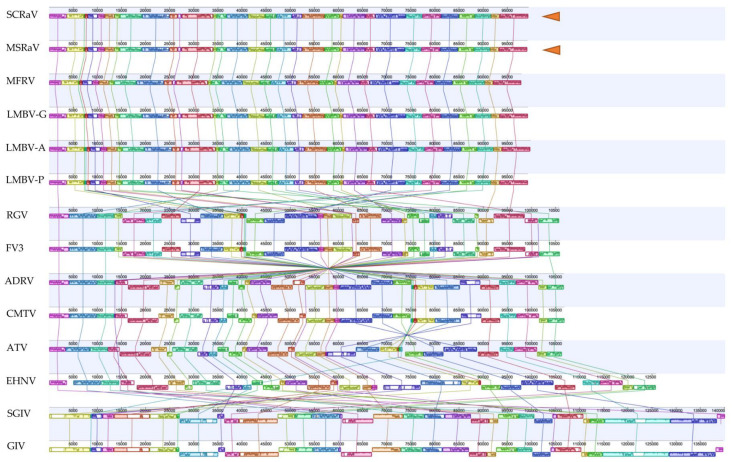
Whole genome alignment of SCRaV, MSRaV, and ranaviruses from other animals. Each genome displays several locally collinear blocks showing in different colored blocks. Related blocks with similar colors and patterns were connected by lines with different colors. There are the following five groups of genomic arrangements: SCRaV and MSRaV-like, RGV and FV3, ADRV and CMTV, ATV and EHNV, SGIV and GIV. The two viruses in the present study are indicated by yellow triangles.

We tried to perform a dot plot analysis to determine the genome similarity degrees between the two viruses and other ranaviruses, but no obvious collinearity can be found, possibly because of the low sequence identity between the two virus genomes and other ranaviruses. Then, a genome-wide alignment was carried out and revealed the genomic arrangement of the aligned ranaviruses (Figure 5). The genome of the 14 ranaviruses can be divided into more than 20 locally collinear blocks (LCBs), which were indicated by different colors in the figure. It can be observed that there were 5 types of genomic arrangement in the aligned ranavirus genomes based on the arrangement of LCBs. All the ranaviruses isolated from mandarin fish and largemouth bass, including SCRaV and MSRaV, have the same genomic arrangement and belong to the first type named SCRaV/MSRaV/LMBV-like or Santee-Cooper ranavirus (SCRV), and RGV and FV3 have the same second type of genomic arrangement. ADRV and CMTV possess the third type of genomic arrangement. ATV and EHNV have the fourth type of genomic arrangement. SGIV and GIV have the fifth type of genomic arrangement. LCBs arrangement of SCRaV/MSRaV/LMBV-like viruses was obviously different from the other four types. For example, the LCB at genome regions of about 75–80 kbp in SCRaV/MSRaV/LMBV-like viruses were located at regions of about 16–23 kbp in RGV and FV3, at regions about 93–101 kbp in ADRV and CMTV, and regions of about 103–111 kbp in ATV. The 3′-end of the genome of the FV3-, CMTV-, and ATV-like viruses all correspond to a central region located at 35–37 kbp of genomes from SCRaV and MSRaV. Arrangement of these LCBs revealed the genomic insertion, inversion, and rearrangement among the ranaviruses and also indicated that SCRaV and MSRaV-like viruses have unique genome arrangements in ranaviruses. Thus, combined with the genome type represented by SGIV and GIV, there are 5 genome types in the sequenced ranaviruses.

## 4. Discussion

Fish ranaviruses are getting more and more attention for the development of the aquaculture industry, such as these infecting fishes of the order Perciformes. However, a detailed analysis of the genome architecture of ranaviruses from Perciformes fish and a comparison with other ranaviruses was lacking. In the present study, based on two newly isolated ranaviruses from mandarin fish and largemouth bass, genome characters of the types of ranaviruses were analyzed.

Sequence comparison showed that there was highly sequence identity between SCRaV and MSRaV, which indicated that the two viruses should belong to one species. Among the eleven proteins that possessed differences between the two viruses, the 79L (predicted neurofilament triplet H1-like protein) of the two viruses had identities lower than 90%, which hinted that the proteins, especially the 79L, could determine the characteristics of the two viruses. We also observed that the proteins among the SCRaV/MSRaV-like viruses isolated in China possessed more sequence identity than that of virus isolates of the USA, and vice versa, especially for six proteins, including a DNA polymerase subunit, which indicated that these proteins may be associated with the regional divergence and replication efficacy of the viruses.

Sequence divergence between the type of ranavirus and other ranaviruses (e.g., FV3/RGV-like, ATV/EHNV-like, CMTV/ADRV-like, and SGIV-like) is relatively high, which indicated that the ranaviruses isolated from mandarin fish and largemouth bass have their own characters. Up to now, reports on gene functions of the type of ranaviruses are few. It could be observed that the MCP of SCRaV and MSRaV have the highest sequence identity with its homolog of other ranaviruses, which indicated the high homology of MCPs among ranaviruses. On the contrary, several proteins possessing low homology with other ranaviruses were found. The viral proteins that could be involved in virus–host interactions all belonged to the low homology proteins, which indicated the adaptation to a specific host.

Genome-wide recombination, deletion, insertion, and inversion have been reported in ranaviruses [6,10,14,30]. Our genome alignment showed the sequence inversion and insertion among different types of ranaviruses. The inversion and insertion may be an adaption of viruses to different hosts or environments, which can be used as the basis to classify different types of ranaviruses and also would help in the identification or prediction of emerging and re-emerging ranaviruses. Combined with the results from sequence identity comparison, genome-wide alignment, and phylogenetic analysis, the SCRaV and MSRaV or SCRV-like viruses constitute a unique type/group in ranaviruses. 

NCLDVs usually encode their own proteins to conduct DNA replication and transcription. Our previous study with ADRV and RGV has revealed the replication and transcription machinery of ranaviruses [27]. For DNA replication, the viral DNA polymerase, helicase/primase, PCNA, and SSB should be key components of the replisome. The four proteins were identified in SCRaV and MSRaV encoded proteins (SCRaV 66R, 37R, 100L, and 12L), which indicated that the core components of the replisome of SCRaV and MSRaV were similar with ranaviruses infecting amphibians. Interestingly, domain/motif search showed that two proteins of SCRaV (77L and 82L) contain domains of DNA polymerase III subunits. DNA polymerase III is the main enzyme in bacterial DNA replication [31]. Whether the two proteins participated in ranavirus DNA replication needs to be researched in the future. For DNA transcription, there are 3 predicted RNAP subunits (45R, 74R, and 28R) and 3 possible transcription factors (22L, 40L, and 7R) in SCRaV-encoded proteins, but the number is lower than the need for a complete RNAP in eukaryotes [32,33]. There should be host factors involved in the genome transcription of SCRaV-like viruses, as occurred in ADRV and RGV [27].

To facilitate virus infection, viruses usually encode multiple proteins to regulate cellular processes [34]. Immune responses are important strategies to resist virus infection. It has been reported that two proteins of ranaviruses, the homolog of RNase III and eIF2α, have the ability to regulate the activation of host interferon responses [35,36,37,38]. The two proteins were both identified in SCRaV encoded proteins (23R and 26R), although the eIF2α homolog of SCRaV only has a sequence identity of about 30% with corresponding homologs of other ranaviruses. Other cellular processes include inflammation and apoptosis. SCRaV encodes homologs of LITAF, TNFR, and apoptosis regulator (61L, 72L, 41L, and 70L), which could have functions in the regulation of cell death and inflammation and prompt virus infection, as reported in other ranaviruses [39,40,41]. Interestingly, SCRaV-like ranavirus was found to encode a homolog of insulin-like growth factor (SCRaV 95L). Its homolog in ranaviruses was only found in SGIV, which could modulate cell proliferation and apoptosis [42]. In vitro synthesized viral insulin-like peptides have activities in mammalian cells [43]. However, its function in SCRaV-like viruses in vivo need to be investigated in the future.

It should be noted that there are 5 predicted proteins containing characters of LPXTG-anchored collagen-like adhesins that are mainly found in Enterococci and function as a virulence factor [44]. Whether they also have a function in viral virulence in SCRaV and MSRaV infection remains unknown up to now.

In conclusion, the present study provided a complete genome analysis for SCRaV/MSRaV/LMBV-like ranaviruses, especially the genome architecture and variations compared with other ranaviruses. These results provided new information for understanding the genetic evolution of ranaviruses from fish species and other animals and also facilitated the early warning of fish ranavirus epidemics.

## Figures and Tables

**Table 1:** Characterization of predicted open reading frames (ORFs) of SCRaV and MSRaV ^a^. ORFs of SCRaV were used as reference (the first column).

ORF/aa	Nucleotide Position	Predicted Function/Conserved Domain	kDa	MSRaV ^c^ (OQ267587)		MFRV ^c^ (MG941005)		LMBV-G ^c^ (MW630113)		LMBV-A ^c^ (MK681855)		LMBV-P ^c^ (MK681856)		ADRV ^c^ (KC865735)		RGV ^c^ (JQ654586)		FV3 ^c^ (AY548484)		EHNV ^c^ (MT510742)		SGIV ^c^ (NC_006549)	
				ORF/AA	%ID ^d^	ORF/AA	%ID ^d^	ORF/AA	%ID ^d^	ORF/AA	%ID ^d^	ORF/AA	%ID ^d^	ORF/AA	%ID ^d^	ORF/AA	%ID ^d^	ORF/AA	%ID ^d^	ORF/AA	%ID ^d^	ORF/AA	%ID ^d^
1L/345 ^b^	1–1038	myristylated membrane protein	37.2	1L/345	100	123L/354	97.5	4L/345	100	1L/354	96.1	1L/354	96.1	2L/325	75.2	2L/323	75.2	2L/320	75.2	1L/350	74.4	19R/342	70.5
2L/290	1044–1916	hypothetical protein	33.2	2L/290	100	124L/290	100	5L/290	100	2L/290	98.6	2L/290	98.6	3L/291	45.1	3L/292	44	3L/279	44	2L/279	44.7	18R/285	36.7
3R/404	1943–3157	hypothetical protein	44.7	3R/404	100	NA/NA	NA	6R/404	100	3R/404	99.5	3R/404	99.5	4R/404	51.4	4R/404	51.2	3R/438	51.2	3R/404	50.8	16L/413	39
4R/253	3184–3945	N-terminal immunoglobulin (Ig)-like domain	27.3	4R/253	100	1R/253	100	7R/253	100	4R/253	98.4	4R/165	98.2	NA/NA	NA	NA/NA	NA	NA/NA	NA	NA/NA	NA	NA/NA	NA
5R/54	3982–4146	TM	6.1	5R/54	100	2R/54	100	NA/NA	NA	NA/NA	NA	NA/NA	NA	5R/60	45.5	5R/60	47.7	4R/60	47.7	4R/60	48.8	15L/59	45.7
6R/141	4187–4612	hypothetical protein	15.5	6R/141	100	3R/141	100	8R/141	100	5R/141	97.9	5R/141	97.9	80L/139	37.6	33R/104	35.4	31R/139	37.6	67R/139	37.6	14L/141	33.1
7R/634	4627–6531	transcription termination factor Rho	67.7	7R/624	98.4	4R/598	94.2	9R/396	89.4	6R/633	96.3	6R/621	94.4	79L/640	51	34R/644	51	32R/629	50	68R/658	49	12L/1024	31.1
8R/62	6581–6769	TM	6.8	8R/62	100	6R/62	100	NA/NA	NA	NA/NA	NA	NA/NA	NA	78L/63	62.1	35R/63	62.1	33R/63	63.8	69R/63	63.8	11L/62	53.2
9R/119	6872–7231	L protein-like protein, TM	12.8	9R/119	100	8R/119	100	11R/99	100	7R/99	99	7R/99	99	77L/106	55.2	36R/106	53.1	34R/106	53.1	70R/107	52.5	9L/154	42.5
10L/163	7277–7768	hypothetical protein	16.2	10L/163	100	9L/166	98.2	12R/244	26.3	54/288	42.9	NA/NA	NA	NA/NA	NA	72R/115	47.7	36L/207	44.8	NA/NA	NA	7L/307	29.5
11R/245	7500–8237	LPXTG-anchored collagen-like adhesin	24.4	11R/245	100	10R/248	98.8	12R/244	98.8	9R/102	46.6	9R/102	46.6	75L/144	54.6	38R/91	39.2	65L/54	54	47L/112	51.4	8L/230	40.5
12L/261	8285–9070	p31K protein	29.3	12L/261	100	11L/261	100	13L/261	100	10L/262	99.2	10L/262	99.2	85L/261	77	27R/261	77	25R/262	77	60R/304	77	6R/259	64.4
13R/374	9190–10,314	hypothetical protein	42.3	13R/374	100	12R/374	100	14R/374	100	11R/374	98.9	11R/374	98.9	NA/NA	NA	NA/NA	NA	NA/NA	NA	NA/NA	NA	4L/365	22.3
14L/354	10,373–11,437	hypothetical protein	39.2	14L/354	100	14L/354	100	15L/354	100	12L/354	99.4	12L/354	99.4	60R/237	65.9	52L/355	64.5	52L/355	64.8	54R/355	64.8	3R/381	55.8
15L/71	11,525–11,740	hypothetical protein	7.6	15L/71	100	NA/NA	NA	NA/NA	NA	NA/NA	NA	NA/NA	NA	NA/NA	NA	NA/NA	NA	NA/NA	NA	NA/NA	NA	NA/NA	NA
16R/503 ^b^	11,706–13,217	myristylated membrane protein	53.1	16R/503	100	17R/503	100	16R/503	99.8	13R/503	99.2	13R/503	99.2	58L/522	63.7	53R/522	63.3	53R/522	63.5	53L/523	63.9	88L/506	55.4
17L/191	13,299–13,874	Tumor necrosis factor receptor	20.5	17L/191	100	19L/191	100	17L/191	100	14L/191	96.3	14L/191	96.3	NA/NA	NA	NA/NA	NA	NA/NA	NA	NA/NA	NA	NA/NA	NA
18R/220	13,940–14,602	DNA methyltransferase	25.4	18R/220	100	20R/220	100	18R/220	100	15R/220	97.7	15R/220	97.7	24L/214	65.3	90R/214	64.8	83R/214	65.3	20L/214	65.3	NA/NA	NA
19R/192	14,587–15,165	Methylase of polypeptide chain release factors	20.5	19R/192	100	21R/177	100	19R/177	100	16R/177	94.9	16R/177	94.9	NA/NA	NA	NA/NA	NA	NA/NA	NA	NA/NA	NA	NA/NA	NA
20R/197	15,271–15,864	hypothetical protein	22	20R/197	100	22R/197	100	20R/197	100	17R/196	97	17R/196	97	NA/NA	NA	NA/NA	NA	NA/NA	NA	NA/NA	NA	NA/NA	NA
21L/147	15,925–16,368	immediate early protein ICP-18	16.4	21L/147	100	23L/147	100	21L/147	100	18L/147	98.6	18L/147	98.6	26L/157	39.9	89R/157	40.5	82R/157	40.5	22L/157	39.2	86R/154	49
22L/91 ^b^	16,431–16,706	transcription elongation factor S-II	10.3	22L/91	100	24L/91	98.9	NA/NA	NA	NA/NA	NA	NA/NA	NA	27L/92	56.3	88R/92	56.3	81R/92	56.3	23L/92	55.2	85R/92	51.7
23R/385 ^b^	16,736–17,893	RNAseIII	42.2	23R/385	100	25R/385	100	22R/385	100	19R/370	99.2	19R/385	99.2	28R/372	60.7	87L/371	61.7	80L/371	61.7	24R/372	61.4	84L/375	52.3
24L/464	17,939–19,333	ATPase-dependent protease	52.4	24L/464	100	26L/464	100	23L/464	99.8	20L/464	99.6	20L/464	99.6	29L/558	52.3	86R/572	52.6	79R/572	52.8	25L/645	52.8	83R/445	41.6
25R/101	19,415–19,720	hypothetical protein	11.3	25R/101	100	27R/101	100	24R/101	100	21R/101	100	21R/101	100	NA/NA	NA	NA/NA	NA	NA/NA	NA	NA/NA	NA	NA/NA	NA
26R/270	19,764–20,576	putative eIF-2 alpha-like protein	30.6	26R/270	100	28R/270	100	25R/270	100	22R/270	98.2	22R/270	97.8	84L/233	30.7	28R/69	31.1	26R/76	31.4	61R/259	30.7	NA/NA	NA
27R/957 ^b^	20,666–23,539	tyrosine kinase	107.2	27R/957	100	29R/957	100	26R/957	100	23R/957	99.7	23R/957	99.7	83L/837	54.6	29R/970	53.6	27R/970	53	62R/970	54.1	78L/790	45.9
28R/159	23,550–24,029	DNA-directed RNA polymerase II subunit	18	28R/159	100	30R/159	100	27R/159	100	24R/159	100	24R/159	100	82L/175	45.1	30R/162	43.8	28R/162	43.8	63R/169	44.1	160L/162	39.7
29R/248	24,149–24,895	capsid maturation protease	27.8	29R/248	100	32R/248	100	28R/248	100	25R/248	99.2	25R/248	99.2	NA/NA	NA	NA/NA	NA	NA/NA	NA	65R/274	27	156L/270	23.9
30R/159	24,994–25,473	hypothetical protein	17.9	30R/159	98.7	33R/159	100	29R/159	100	26R/158	96.6	26R/158	96.6	81R/98	32.1	31L/98	32.7	29L/98	33.7	66R/161	36.3	158L/138	38.2
31L/173	25,518–26,039	hypothetical protein	19.1	31L/173	100	34L/173	100	30L/173	100	27L/114	89.3	27L/173	99.4	50L/184	67.4	61R/184	67.4	NA/NA	NA	NA/NA	NA	157R/174	59.2
32L/240	26,072–26,794	LPXTG-anchored collagen-like adhesin Scl2/SclB	22.5	32L/240	100	35L/240	99.6	31L/240	99.6	28L/240	97.5	28L/240	97.1	75L/144	36.7	NA/NA	NA	65L/54	51.1	40R/240	40.5	56R/246	35.8
33L/257	26,802–27,575	LPXTG-anchored collagen-like adhesin Scl2/SclB	24.8	33L/257	100	36L/257	100	32L/257	99.6	29L/257	98.8	29L/257	98.8	75L/144	35.1	38R/91	45	65L/54	55.1	39R/183	40.8	45L/242	36.1
34L/177	27,575–28,108	hypothetical protein	18.7	34L/177	100	37L/177	100	33L/177	100	30L/177	93.8	30L/177	93.2	NA/NA	NA	NA/NA	NA	NA/NA	NA	NA/NA	NA	NA/NA	NA
35L/223 ^b^	28,163–28,834	hypothetical protein	25.7	35L/223	100	38L/223	100	34L/223	100	31L/223	100	31L/223	100	89R/219	72.9	23L/219	72.9	21L/219	72.9	86R/219	72.4	54R/215	68.9
36R/563	28,859–30,550	hypothetical protein	63.8	36R/563	100	39R/563	100	35R/539	100	32R/539	98.7	32R/539	98.7	NA/NA	NA	NA/NA	NA	NA/NA	NA	NA/NA	NA	NA/NA	NA
37R/955 ^b^	30,673–33,540	hypothetical protein	107.5	37R/955	99.9	40R/955	99.8	36R/955	99.6	33R/955	99.5	33R/955	99.5	88L/975	75	24R/975	75.2	22R/973	76.1	85L/973	76	52L/968	68
38R/70	33,573–33,785	TM	7.7	38R/70	100	42R/70	100	NA/NA	NA	NA/NA	NA	NA/NA	NA	99L/70	52.5	12R/70	52.5	11R/70	54.2	96L/70	52.5	103R/97	38.8
39L/297 ^b^	33,832–34,725	hypothetical protein	33	39L/297	100	44L/297	100	37L/297	100	34L/297	99.7	34L/297	99.7	98R/297	65.3	13L/297	66	12L/297	65.7	95R/297	66.7	118R/319	59.7
40L/253 ^b^	34,770–35,531	replicating factor	29.6	40L/253	100	45L/253	99.6	38L/253	99.2	35L/253	99.6	35L/253	99.6	1R/256	61.5	1R/256	61.5	1R/256	61.9	100R/256	62.4	116R/258	53.5
41L/171	35,589–36,104	myeloid cell leukemia protein	18.7	41L/171	100	46L/171	100	39L/150	100	36L/150	99.3	36L/150	99.3	101R/137	30.6	105R/137	30.8	97R/137	30.8	99R/137	30.5	115R/152	24.5
42L/226	36,107–36,787	hypothetical protein	24.7	42L/226	100	47L/226	100	40L/214	100	37L/214	99.1	37L/214	99.1	100R/228	39.6	104R/223	40.5	96R/223	39.6	98R/228	40.5	111R/255	41
43L/78	36,866–37,102	TM	8.7	43L/78	100	48L/78	100	NA/NA	NA	NA/NA	NA	NA/NA	NA	NA/NA	NA	NA/NA	NA	NA/NA	NA	NA/NA	NA	NA/NA	NA
44L/103	37,104–37,415	TM	11	44L/103	100	49L/103	100	38L/253	100	38L/103	100	38L/103	100	NA/NA	NA	NA/NA	NA	NA/NA	NA	NA/NA	NA	NA/NA	NA
45R/1354^b^	37,201–41,265	DNA-dependent RNA polymerase a subunit	147.1	45R/1354	100	50R/1354	99.9	41R/1353	99.9	39R/1263	99.7	39R/1263	99.7	9R/1294	64.9	9R/1294	64.7	8R/1293	64.8	7R/1303	64.9	104L/1268	62.4
46R/69	41,298–41,507	hypothetical protein	6.2	46R/55	100	NA/NA	NA	NA/NA	NA	NA/NA	NA	NA/NA	NA	NA/NA	NA	NA/NA	NA	NA/NA	NA	NA/NA	NA	NA/NA	NA
47R/136	41,525–41,935	hypothetical protein	14.9	47R/136	100	51R/136	100	NA/NA	NA	NA/NA	NA	NA/NA	NA	55L/379	52.6	57R/379	51.6	55R/379	52.4	NA/NA	NA	NA/NA	NA
48L/401	41,582–42,787	helicase-like protein	44.6	48L/400	100	52L/401	100	42L/401	100	40L/401	98.5	40L/401	98.5	54R/431	55	56L/431	55.2	55L/431	55	51R/431	54.7	152R/412	48
49L/49	42,794–42,943	TM	5.2	49L/49	100	NA/NA	NA	NA/NA	NA	NA/NA	NA	NA/NA	NA	NA/NA	NA	NA/NA	NA	NA/NA	NA	NA/NA	NA	NA/NA	NA
50R/136	42,982–43,392	hypothetical protein	15.5	50R/136	100	53R/136	100	43R/136	100	41R/136	98.5	41R/136	98.5	52L/134	37.8	59R/134	38.6	NA/NA	NA	49L/134	37.8	151L/195	39.3
51R/492 ^b^	43,401–44,879	Serine/threonine protein kinases	53.8	51R/492	100	54R/492	99.8	44R/492	99.8	42R/492	99.2	42R/492	99.2	51L/498	45.2	60R/498	45.6	57R/498	45.6	48L/498	46.3	150L/508	35.4
52L/173	44,939–45,460	hypothetical protein	19.6	52L/173	100	56L/173	100	45L/172	100	43L/172	99.4	43L/172	99.4	NA/NA	NA	NA/NA	NA	NA/NA	NA	33L/160	49	148R/159	40.8
53R/60	45,433–45,615	hypothetical protein	6.5	53R/60	100	57R/60	100	NA/NA	NA	NA/NA	NA	NA/NA	NA	NA/NA	NA	NA/NA	NA	NA/NA	NA	NA/NA	NA	NA/NA	NA
54L/71	45,634–45,849	hypothetical protein	7.9	54L/71	100	58L/71	100	NA/NA	NA	NA/NA	NA	NA/NA	NA	NA/NA	NA	NA/NA	NA	NA/NA	NA	NA/NA	NA	NA/NA	NA
55L/123	45,885–46,256	hypothetical protein	13.7	55L/123	100	59L/123	100	46L/123	100	44L/123	99.2	44L/123	99.2	38L/124	40	77R/124	39.2	70R/124	40	35L/122	41.1	NA/NA	NA
56L/79	46,264–46,503	TM	8.5	56L/79	100	60L/79	100	NA/NA	NA	NA/NA	NA	NA/NA	NA	39L/88	64.8	76R/88	63.4	69R/88	63.4	36L/88	63.4	143L/79	51.9
57R/216	46,591–47,241	hypothetical protein	24	57R/216	100	61R/216	100	47R/216	99.5	45R/216	100	45R/216	100	40R/107	41.1	75L/88	43.1	NA/NA	NA	37R/234	35.9	145R/165	20.5
58L/324	47,338–48,312	NTPase helicase-like protein	36.1	58L/324	100	63L/324	100	48L/324	100	46L/324	99.7	46L/324	99.7	35R/350	44.3	80L/324	44.6	73L/324	44.6	31R/324	44.9	146L/324	47.2
59L/405	48,433–49,650	hypothetical protein	44.6	59L/405	100	65L/405	100	49L/405	100	47L/405	100	47L/405	100	NA/NA	NA	NA/NA	NA	NA/NA	NA	NA/NA	NA	147L/344	24.5
60L/377	49,677–50,810	hypothetical protein	41.3	60L/377	100	67L/377	100	50L/377	100	48L/377	98.4	48L/377	98.4	34R/447	41.8	81L/364	42.7	74L/370	42.3	30R/393	64.6	137R/461	41.1
61L/85	50,859–51,116	lipopolysaccharide-induced TNF-alpha factor-like protein	9.5	61L/85	100	68L/85	100	NA/NA	NA	NA/NA	NA	NA/NA	NA	33R/84	67.1	82L/84	66.3	75L/84	65.9	NA/NA	NA	136R/104	66.7
62R/73	51,175–51,396	hypothetical protein	8.2	62R/73	100	69R/73	100	NA/NA	NA	NA/NA	NA	NA/NA	NA	32L/73	60.3	83R/73	58.9	76R/73	58.9	28L/73	60.3	119R/83	30.4
63L/208	51,446–52,072	hypothetical protein	23.7	63L/208	100	70L/208	100	51L/208	100	49L/208	98.6	49L/208	98.6	30R/212	39.1	85L/224	39.5	78L/212	39.5	26R/255	38.6	122L/210	29.1
64L/371	52,139–53,254	hypothetical protein	42.6	64L/371	100	71L/371	100	52L/371	100	50L/371	99.5	50L/371	99.2	48R/352	30.9	62L/352	32	59L/352	32	45R/352	32.3	123L/362	30.5
65R/182	53,321–53,869	hypothetical protein	20.4	65R/182	100	72R/182	100	53R/182	100	51R/182	98.9	51R/182	98.9	NA/NA	NA	NA/NA	NA	NA/NA	NA	NA/NA	NA	126R/185	44.4
66R/1004 ^b^	53,929–56,943	DNA polymerase	113.7	66R/1004	100	74R/1004	100	54R/1004	100	52R/1004	99.8	52R/1004	99.8	47L/1013	75.8	63R/1013	75.9	60R/1013	75.8	44L/1013	75.6	128R/109	68.8
67L/243	57,000–57,731	LPXTG-anchored collagen-like adhesin Scl2/SclB	23.4	67L/243	100	75L/243	100	55L/238	100	53L/238	97.5	53L/238	97.5	75L/144	42.9	38R/91	55.6	NA/NA	NA	47L/112	46.4	55R/240	59.6
68L/288	57,737–58,603	LPXTG-anchored collagen-like adhesin Scl2/SclB	28.9	68L/288	99.7	76L/288	100	56L/288	100	54L/288	96.5	54L/173	89.5	75L/144	40	38R/91	56.3	NA/NA	NA	39R/183	58.8	112R/355	53.1
69L/387 ^b^	58,652–59,815	ribonucleotide reductase beta subunit	44.1	69L/387	100	77L/387	100	57L/387	100	55L/387	100	55L/387	100	42R/387	77.9	73L/387	77.7	67L/387	77.7	38R/387	77.7	47L/384	74.9
70L/91	59,932–60,207	caspase recruitment domain protein	10.2	70L/91	100	78L/91	100	NA/NA	NA	NA/NA	NA	NA/NA	NA	43L/95	39.8	68R/95	42.1	64R/95	42.1	41L/95	42.1	48L/91	37.7
71L/141	60,269–60,694	deoxyuridine 5’-triphosphate nucleotidohydrolase	15.1	71L/141	99.3	80L/141	100	58L/141	100	56L/141	100	56L/141	100	44L/164	57.3	67R/164	57.3	63R/164	57.3	42L/164	57.3	49L/155	57.9
72L/237	60,795–61,508	tumor necrosis factor receptor, TM	25.4	72L/237	100	81L/237	100	59L/237	100	57L/237	99.6	57L/237	99.6	NA/NA	NA	NA/NA	NA	NA/NA	NA	NA/NA	NA	51L/231	36.7
73L/178	61,588–62,124	hypothetical protein	20	73L/178	100	83L/178	100	60L/178	100	58L/178	99.4	58L/178	99.4	45L/178	41.5	66R/178	40.6	NA/NA	NA	NA/NA	NA	75R/178	38.9
74R/1094 ^b^	62,188–65,472	DNA-dependent RNA polymerase II	120.8	74R/1094	100	84R/1094	100	61R/1094	100	59R/1094	99.6	59R/1094	99.6	46R/1221	70.4	65L/1221	69.9	62L/1221	69.9	43R/1227	70	73L/1103	65.8
75L/356 ^b^	65,516–66,586	DNA repair enzyme RAD2	40	75L/356	100	85L/356	100	62L/356	100	60L/356	98.9	60L/356	98.9	12L/363	59.9	102R/363	59.9	95R/363	59.9	10L/364	59.5	97L/382	59.4
76R/154 ^b^	66,655–67,119	hypothetical protein	17.7	76R/154	100	86R/154	100	63R/154	99.4	61R/154	100	61R/154	100	13R/155	76.6	101L/155	76.6	94L/155	76.6	11R/155	76.6	98R/267	67.6
77L/284	67,653–68,507	DNA polymerase III subunits gamma and tau	29.1	77L/284	100	89L/284	100	64L/284	99.7	62L/263	91.1	62L/263	91.4	67R/290	33	45L/383	37.3	42L/85	34.7	78L/285	33	20L/322	35.9
78L/136	68,563–68,973	hypothetical protein	15.6	78L/136	100	9L/136	100	65L/136	100	63L/136	98.5	63L/136	98.5	66R/136	62.9	46L/136	62.1	45L/136	62.1	NA/NA	NA	21L/139	60.3
79L/287	69,019–69,882	neurofilament triplet H1-like protein	30.8	79L/197	86	92L/191	86	66L/136	86	64L/311	97	64L/239	82.6	65R/169	53.4	47L/144	52.9	46L/81	37.1	80L/203	55.3	22L/166	44.3
80L/136	69,916–70,326	hypothetical protein	16	80L/136	99.3	93L/136	98.5	67L/136	98.5	65L/136	99.3	65L/136	99.3	64R/138	36	48L/138	38.1	47L/138	38.1	81L/138	34.5	24L/151	27.9
81L/73	70,366–70,587	hypothetical protein	8.6	81L/73	100	94L/73	100	NA/NA	NA	NA/NA	NA	NA/NA	NA	NA/NA	NA	NA/NA	NA	NA/NA	NA	NA/NA	NA	NA/NA	NA
82L/566	70,628–72,328	SAP domain-containing protein	61.7	82L/602	92.5	95L/604	93.2	68L/320	90.4	66L/560	88.6	67L/383	95.6	62R/508	48.8	50L/499	47.7	49L/249	49.2	83L/541	50.8	25L/510	54.5
83R/566	72,401–74,101	hypothetical protein	63.2	83R/566	100	96R/566	100	70R/566	100	67R/566	99.3	68R/566	99.1	61L/561	46.2	51R/561	46.6	51R/561	46.6	84R/561	46.6	26R/566	39.9
84L/144	74,152–74,586	hypothetical protein	15.8	84L/144	100	97L/144	100	71L/144	100	68L/144	100	69L/144	100	90L/148	60.1	22R/172	60.8	20R/148	60.1	88L/149	61.8	38L/170	44.7
85L/879 ^b^	74,614–77,253	TM	96.3	86R/113	99.9	99R/113	100	NA/NA	NA	NA/NA	NA	NA/NA	NA	91L/960	41.6	21R/852	41.8	19R/851	40.4	89L/907	41.6	39L/1051	38.7
86R/113	76,955–77,296	2-cysteine adaptor domain protein	12	85L/879	100	98L/879	99.9	72L/879	99.9	69L/879	99.2	70L/879	99.3	92R/78	42.4	20L/79	46.2	18L/78	48.5	NA/NA	NA	NA/NA	NA
87R/503	77,329–78,840	hypothetical protein	54.3	87R/503	100	100R/503	100	73R/503	100	70R/503	99.8	71R/503	99.8	93R/502	54.5	19L/502	54.9	17L/502	55.1	90R/502	54.7	43R/667	39.3
88R/281	78,924–79,769	hypothetical protein	31	88R/281	100	101R/281	100	74R/281	100	71R/281	100	72R/281	100	95L/216	45.7	17R/275	46.7	16R/275	46.1	91R/291	47.4	132R/275	39.7
89L/315 ^b^	79,982–80,929	ABC-ATPase	35.8	89L/315	100	103L/293	100	75L/293	100	72L/300	99.3	73L/300	99.3	96L/315	80.9	16R/315	80.9	15R/322	80.9	92L/308	80.9	134L/323	71.2
90L/119	80,896–81,255	hypothetical protein	13.7	90L/119	100	104L/119	100	76L/119	100	73L/119	99.2	74L/119	99.2	97L/119	52.1	15R/84	60.8	14R/119	52.1	93L/118	52.1	135L/112	46.7
91L/1162 ^b^	81,325–84,813	TM	129.7	91L/1162	100	105L/1162	100	77L/1162	99.9	74L/1162	99.8	75L/1162	99.8	68L/1165	60	44R/1165	60.1	41R/1165	59.8	77R/1165	60	57L/1168	52
92L/91	84,867–85,142	TM	10.2	92L/91	100	107L/91	100	NA/NA	NA	NA/NA	NA	NA/NA	NA	NA/NA	NA	NA/NA	NA	40R/128	44	75R/268	44	NA/NA	NA
93L/106	85,201–85,521	hypothetical protein	12.3	93L/106	100	108L/106	100	78L/101	100	75L/106	100	76L/106	100	NA/NA	NA	NA/NA	NA	NA/NA	NA	NA/NA	NA	NA/NA	NA
94L/562	85,585–87,273	ribonucleotide reductase alpha subunit	62.9	94L/562	100	109L/562	99.8	79L/562	100	76L/562	99.6	77L/562	99.6	71L/565	78.8	41R/565	78.5	38R/565	78.1	73R/254	78.9	64R/572	70.3
95L/79	87,364–87,603	insulin-like growth factor	8.4	95L/79	100	110L/79	100	NA/NA	NA	NA/NA	NA	NA/NA	NA	NA/NA	NA	NA/NA	NA	NA/NA	NA	NA/NA	NA	62R/256	38.7
96L/205 ^b^	87,643–88,260	NIF/NLI interacting factor	23.4	96L/205	100	111L/205	100	80L/205	100	77L/205	99.5	78L/205	99.5	72L/213	59.2	40R/213	59.2	37R/209	59.2	72R/211	59.2	61R/204	50.5
97L/949 ^b^	88,278–91,127	NTPase	106.4	97L/949	100	112L/949	100	81L/949	100	78L/949	99.4	79L/949	99.4	10L/948	68.2	10L/948	68.2	9L/948	68.2	8L/948	68.1	60R/970	60.6
98R/132	91,149–91,547	TM	14.6	98R/132	100	113R/132	100	82R/132	100	79R/132	100	80R/132	100	11R/137	46	11R/137	43.1	10R/137	43.1	9R/137	47.5	59L/146	32.4
99L/189 ^b^	91,617–92,186	deoxyribonucleoside kinase	20.8	99L/189	100	115L/189	100	83L/189	100	80L/189	99.5	81L/189	99.5	22L/195	52.7	92R/195	52.2	85R/195	52.2	18L/195	55.2	67L/191	53.6
100L/242 ^b^	92,238–92,966	hypothetical protein	25.8	100L/242	100	116L/242	100	84L/242	100	81L/242	99.2	82L/242	99.2	23L/245	60.5	91R/245	61.3	84R/245	60.9	19L/260	60.9	68L/272	50
101L/594	93,049–94,833	hypothetical protein	66.4	101L/594	99.8	117L/594	100	85L/594	100	82L/594	99.5	83L/594	99.5	20R/605	34.9	94L/593	33.6	87L/605	34.9	17R/617	34.6	69L/548	32
102R/147 ^b^	94,867–95,310	thiol oxidoreductase	16.6	102R/147	100	118R/147	100	86R/147	100	83R/147	99.3	84R/147	99.3	19L/150	62.3	95R/150	61.6	88R/150	61.6	16L/150	61	70R/152	54.6
103R/413	95,316–96,557	hypothetical protein	48.5	103R/398	96.4	119R/368	88.9	1R/387	93.7	84R/390	81.8	85R/390	83.8	18L/414	36	96R/381	37.4	89R/388	38.1	15L/368	37.1	71R/274	38.8
104R/463 ^b^	96,670–98,061	major capsid protein	50.1	104R/463	100	120R/463	100	2R/463	100	85R/463	99.4	86R/463	99.4	17L/463	84	97R/463	83.6	90R/463	83.2	14L/463	83.4	72R/463	73.7
105R/382 ^b^	98,174–99,322	immediate early protein ICP-46	43.9	105R/382	100	121R/382	100	3R/382	100	86R/382	99.7	87R/382	99.7	16L/395	57.1	98R/395	57.6	91R/395	57.9	13L/395	57.6	162L/382	50.7

^a^ TM, transmembrane domain; aa, number of amino acids of each protein; kDa, molecular mass of each protein as predicted by Detaibio website tools; ID, identity; NA, not annotated (denotes no corresponding homologous ORF in the genome). MFRV, mandarin fish ranavirus; LMBV-G, largemouth bass virus strain GDOU; LMBV-A, largemouth bass virus strain Alleghany; LMBV-P, largemouth bass virus strain Pine; ADRV, *Andrias davidianus ranavirus*; RGV, *Rana grylio* virus; FV3, frog virus 3; EHNV, epizootic hematopoietic necrosis virus; SGIV, Singapore grouper iridovirus. ^b^ Core genes of iridoviruses. ^c^ Corresponding homologous ORFs in the indicated virus genomes based on BLASTP analysis. ^d^ Amino acid identities were calculated using the ClustaW method in the MegAlign program.

## Data Availability

The complete genome sequence of SCRaV and MSRaV have been submitted into NCBI GenBank. The accession number of SCRaV is OQ267588, and MSRaV is OQ267587. Data is contained within the article or supplementary material. The raw data is available upon reasonable request from the corresponding author.

## Supplementary Materials

The following supporting information can be downloaded at: https://www.mdpi.com/article/10.3390/pathogens12050730/s1, Table S1: Virus name and accession number, Table S2: Information of six ranavirus isolates from *Siniperca chuatsi* and *Micropterus salmoides*.
