# Supplementary material for: Genome Characteristics of Two Ranavirus Isolates from Mandarin Fish and Largemouth Bass
Source: Pathogens. 2023 May 17;12(5):730. doi: 10.3390/pathogens12050730 (PMC10223008; doi:10.3390/pathogens12050730)
Supplement: Supplementary file 1 [file pathogens-12-00730-s001.zip › Table S1 virus name and accession number-R1.pdf]

Table S1. Full name, abbreviation, and GenBank accession number of the 56 viruses used in the study.

**Genus**	**Virus name (Abbreviation)**	**Genome size(bp)**	**GC%**	**Accession number**
Ranavirus	*Rana grylio* iridovirus (RGV)	105791	55.07	JQ654586
	Soft-shelled turtle iridovirus	105890	55.06	EU627010
	Rana nigromaculata ranavirus	104286	55.17	MG791866
	Frog virus 3 (FV3)	105903	55.05	AY548484
	Frog virus 3 strain Rana-Bra	104744	54.94	MT578298
	Tiger frog virus	105057	55.01	AF389451
	Bohle iridovirus	103531	55.16	KX185156
	German gecko ranavirus	103681	55.06	KP266742
	Zoo ranavirus	103266	55.05	MK227779
	Tortoise ranavirus	103876	55.22	KP266743
	Common midwife toad virus-E (CMTV)	106878	55.25	JQ231222
	Pike-perch iridovirus	108041	55.29	KX574341
	Pelophylax esculentus virus	107469	55.18	MF538627
	Red-eared slider ranavirus	106878	55.35	MT452035
	Common midwife toad virus isolate-Pe	107054	55.24	MF125269
	Testudo hermanni ranavirus	105811	55.36	KP266741
	Rana catesbeiana virus	106890	54.98	KX397571
	Andrias davidianus ranavirus (ADRV)	106734	55.02	KC865735
	Chinese giant salamander iridovirus	105375	55.22	KF512820
	Lumpfish ranavirus	115947	54.68	MH665359
	Cod iridovirus	114865	54.92	KX574342
	Ranavirus maximus	115510	54.94	KX574343
	Epizootic haematopoietic necrosis virus (EHNV)	125860	54.18	MT510742
	European catfish virus	127549	54.25	KT989885
	European sheatfish virus	127732	54.23	JQ724856
	Ambystoma tigrinum stebbensi virus (ATV)	106332	54.02	AY150217
	Short-finned eel ranavirus	126965	54.71	KX353311
	Largemouth bass virus strain Alleghany (LMBV-A)	99827	51.93	MK681855
	Largemouth bass virus strain Pine (LMBV-P)	99290	51.91	MK681856
	Largemouth bass virus strain GDOU (LMBV-G)	99315	52.08	MW630113
	Mandarin fish ranavirus (MFRV)	97946	52.10	MG941005
	Micropterus salmoides Ranavirus (MSRaV)	99171	52.09	OQ267587
	Siniperca Chuatsi Ranavirus (SCRaV)	99405	52.09	OQ267588
	Grouper iridovirus	139793	48.60	AY666015
	Singapore grouper iridovirus (SGIV)	140131	48.64	NC_006549
Megalocytivirus	European chub iridovirus	128216	38.40	MK637631
	Scale drop disease virus	131129	36.55	MN562489
	Turbot reddish body iridovirus	110104	54.99	GQ273492
	Infectious spleen and kidney necrosis virus isolate EFIV	111380	54.76	MW273354
	Infectious spleen and kidney necrosis virus	111362	54.78	NC_003494
	Large yellow croaker iridovirus	112043	53.47	MW139932
	Rock bream iridovirus strain RBIV-KOR-TY1	112080	53.03	AY532606
	Rock bream iridovirus isolate RBIV-C1	112333	53.00	KC244182
	Red seabream iridovirus	111557	53.03	MT798582

	Giant sea perch iridovirus	112565	53.02	KT804738
	Orange-spotted grouper iridovirus	112636	53.03	AY894343
Lymphocystivirus	Lymphocystis disease virus 1	102653	29.07	NC_001824
	Lymphocystis disease virus Sa	208501	33.00	NC_033423
	Lymphocystis disease virus isolate China	186250	27.23	AY380826
	Lymphocystis disease virus 4	211086	26.03	MN803438
Decapodiridovirus	*Cherax quadricarinatus* iridovirus	165695	34.57	NC_040612
	Shrimp hemocyte iridescent virus	165809	34.58	MF599468
Chloriridovirus	*Aedes taeniorhynchus* iridescent virus	191100	47.89	NC_008187
	Invertebrate iridovirus 25	204815	30.32	HF920635
Iridovirus	*Armadillidium vulgare* iridescent virus	220222	35.08	NC_024451
	Invertebrate iridescent virus 6	212482	28.62	NC_003038
