# Supplementary material for: Genome Characteristics of Two Ranavirus Isolates from Mandarin Fish and Largemouth Bass
Source: Pathogens. 2023 May 17;12(5):730. doi: 10.3390/pathogens12050730 (PMC10223008; doi:10.3390/pathogens12050730)
Supplement: Supplementary file 1 [file pathogens-12-00730-s001.zip › Table S2 Comparison of 6 ranavirus in Siniperca Chuatsi and Micropterus salmoides.pdf]

Table S2. Comparison of 6 ranavirus in Siniperca Chuatsi and Micropterus salmoides

Virus name	Nucleotide size (bp)	Identity%[a]	CG%	Location	host
SCRaV	99405	100	52.09	China Hubei	Mandarin fish (*Siniperca chuatsi*)
MSRaV	99171	99.92	52.09	China Hubei	Largemouth bass (*Micropterus salmoides*)
LMBV-A	99827	98.7	51.93	America Pennsylvania	Largemouth bass (*Micropterus salmoides*)
LMBV-P	99290	98.68	51.9	America Pennsylvania	Largemouth bass (*Micropterus salmoides*)
LMBV-G	99315	99.82	52.09	China Guangdong	Largemouth bass (*Micropterus salmoides*)
MFRV	97946	99.88	52.11	China Jiangsu	Mandarin fish (*Siniperca chuatsi*)

[a.] The value of identities was product by BLASTN each compared to SCRaV.
